# Safety and tolerability of linezolid to treat tuberculosis: a systematic review and meta-analysis

**DOI:** 10.36416/1806-3756/e20250464

**Published:** 2026-08-13

**Authors:** Mehrnaz Amiri, Mahdis Cheraghi, Nasim Ghafari, Mohammad Javad Nasiri, Denise Rossato Silva, Lia D’Ambrosio, Rosella Centis, Simone Villa, Ananthu James, Emanuele Pontali, Giovanni Battista Migliori

**Affiliations:** 1. Department of Microbiology, School of Medicine, Shahid Beheshti University of Medical Sciences, Tehran, Iran.; 2. Faculdade de Medicina, Universidade Federal do Rio Grande do Sul - UFRGS - Porto Alegre (RS) Brasil.; 3. Public Health Consulting Group, Lugano, Switzerland.; 4. Servizio di Epidemiologia Clinica delle Malattie Respiratorie, Istituti Clinici Scientifici Maugeri - IRCCS - Tradate, Italia.; 5. Dipartimento di Scienze Biomediche per la Salute, Università degli Studi di Milano, Milano, Italia.; 6. mDrift Technologies, Rotterdam, The Netherlands; 7. S. C. Malattie Infettive, Ospedali Galliera, Genova, Italia.

**Keywords:** Linezolid, Tuberculosis, pulmonary, Tuberculosis, multidrug-resistant, drug tolerance, adverse drug reactions

## Abstract

**Objective:**

In a previous review, we summarized the safety of linezolid-containing regimens to treat rifampin-resistant/multidrug-resistant tuberculosis (RR/MDR-TB), underscoring the persistent need for shorter and safer regimens. The objective of the present study was to explore the safety and tolerability directly attributable to linezolid in regimens designed to treat RR/MDR-TB in studies published between 2009 and 2025.

**Methods:**

We searched PubMed/MEDLINE, Embase, the Cochrane Central Register of Controlled Trials, Scopus, and Web of Science for studies reporting on the safety and tolerability of linezolid since it was introduced as a repurposed drug in the treatment of tuberculosis.

**Results:**

A total of 22 studies (including 26 arms) were identified, with a total of 3,014 patients. There was significant variation in geographic distribution, sample size (range, 8-655), linezolid dose (range, 300-1,200 mg), duration, and other core variables. The pooled rate of favorable outcomes among cohort studies (cure or treatment completion) was = 80.1%. Hematologic disorders (including anemia, thrombocytopenia, and leukopenia, in 33.1%) and peripheral neuropathy (in 33.9%) were the most common adverse events (AEs), followed by psychiatric and neurological disorders (in 12.3%), myalgia (in 7.0%), optic neuropathy (in 7.8%), gastrointestinal events (in 1.9%), ototoxicity (in 2.9%), and rash (in 1.0%). Serious AEs were rarely reported.

**Conclusions:**

Although the recommended linezolid-containing regimens are considered safe, linezolid still causes frequent and severe AEs. A new drug with similar characteristics but lower toxicity will significantly improve the safety of antituberculosis regimens, with improved adherence and treatment outcomes.

## INTRODUCTION

With an estimated 10.7 million people suffering from tuberculosis, a tuberculosis incidence rate (new cases per 100,000 population per year) of 131, 1.23 million deaths from the disease, and 8.3 million people newly diagnosed with tuberculosis as reported by the 2024 WHO Global TB Report, tuberculosis continues to be a first-class clinical and public health priority worldwide. 

According to 2024 WHO estimates, a total of 164,545 people were treated for rifampin-resistant tuberculosis (RR-TB), this being 42% of the approximately 390,000 people who developed RR-TB or multidrug-resistant tuberculosis (RR/MDR-TB) in 2024, almost the same as in 2023.^1^

It is well known that both clinical and programmatic management of RR/MDR-TB (which includes pre-extensively drug-resistant and extensively drug-resistant tuberculosis-XDR-TB) poses additional challenges in comparison with treating tuberculosis forms that are susceptible to the drugs,^1^ i.e., establishing a rapid and accurate diagnosis requires high-quality laboratories, and designing effective regimens is more complex. The new and repurposed drugs recommended by the WHO are, in fact, associated with more frequent and often severe adverse events (AEs).^2^ Among the WHO group A drugs (including fluoroquinolones, bedaquiline, and linezolid), linezolid has demonstrated relevant toxicity, similar to that of other drugs in WHO groups B and C.^2^ ^,^ ^3^

Although increasing evidence supports the effectiveness of current regimens for treating RR/MDR-TB,^1^ ^,^ ^4^ ^-^ ^6^ the safety and tolerability profiles of such regimens have been less systematically characterized.^2^ ^,^ ^7^ ^,^ ^8^ Beyond a recent scoping review that broadly evaluated the safety and tolerability of RR/MDR-TB regimens, including those containing linezolid,^9^ several studies have reported AEs attributable to the regimens as a whole, whereas evidence on the frequency, severity, and clinical impact of AEs caused by linezolid itself is scanty. 

The objective of the present systematic review and meta-analysis was to explore the safety and tolerability directly attributable to linezolid in regimens designed to treat RR/MDR-TB in studies published between 2009 and 2025. 

## METHODS

### Study design

We performed a systematic review to identify cohort studies and randomized controlled trials (RCTs) reporting on AEs attributable to linezolid in patients with pulmonary RR/MDR-TB. The review was conducted and reported in accordance with the 2020 Preferred Reporting Items for Systematic Reviews and Meta-Analyses statement and was registered with the International Prospective Register of Systematic Reviews (PROSPERO ID: 1164134). 

A comprehensive literature search of PubMed/MEDLINE, Embase, the Cochrane Central Register of Controlled Trials, Scopus, and Web of Science was performed for records published between January 1, 2009 and June 20, 2025. The complete search strategies for all databases are provided in the supplementary material (Chart S1). 

### Definitions

MDR-TB was defined as *Mycobacterium tuberculosis* resistant to at least the two core first-line antituberculosis drugs, i.e., isoniazid and rifampin.^1^

According to the 2013 WHO classification,^10^ XDR-TB referred to MDR-TB with additional resistance to any fluoroquinolone and/or at least one of the three second-line injectable agents (kanamycin, amikacin, or capreomycin). 

Under the revised 2021 WHO definitions,^11^ pre-XDR-TB is defined as MDR-TB with additional resistance to any fluoroquinolone, whereas XDR-TB is defined as tuberculosis caused by *M. tuberculosis* strains resistant to a fluoroquinolone and at least one additional group A drug (linezolid or bedaquiline), indicating resistance to two of the three group A drugs. 

### Study selection

All retrieved records were imported into EndNote X8 (Thomson Reuters, Toronto, ON, Canada) for de-duplication. Two reviewers (MA and MC) independently screened titles and abstracts, followed by full-text evaluation of potentially eligible studies. Discrepancies were resolved through discussion or consultation with a third reviewer (MJN). 

Eligible studies included cohort studies and RCTs or nonrandomized controlled trials evaluating AEs directly attributable to linezolid in adults with confirmed pulmonary drug-resistant tuberculosis, including RR-TB, MDR-TB, pre-XDR-TB, and XDR-TB. Only studies involving linezolid-containing regimens were included. 

We excluded reviews, case reports, case series, cross-sectional studies, case-control studies, conference abstracts, and studies focused exclusively on children or pregnant women. Studies involving extrapulmonary tuberculosis or lacking clear attribution of AEs to linezolid exposure were also excluded. 

### Data extraction

The same two reviewers independently extracted data using a standardized Microsoft Excel spreadsheet (Microsoft Corp., Redmond, WA, USA). Discrepancies were resolved by consensus or consultation with the third reviewer. Extracted data included study characteristics (first author, publication year, design, period, country, and setting), participant demographics, treatment details (total regimen duration, duration of linezolid exposure, and number of patients receiving linezolid), and clinical outcomes. Reported linezolid-attributed AEs and the distribution of drug-resistance categories (RR-TB, MDR-TB, pre-XDR-TB, and XDR-TB) were also recorded.

### Quality assessment

The same two reviewers independently assessed the methodological quality of the included studies, with disagreements being resolved by the third reviewer. The Newcastle-Ottawa Scale was applied to observational studies, evaluating selection, comparability, and outcome domains, and studies were categorized as high quality (7-9), moderate quality (4-6), or low quality (0-3). RCTs were evaluated by means of the Cochrane Risk of Bias tool, which examines random sequence generation, allocation concealment, blinding, completeness of outcome data, and selective reporting. 

### Data analysis

All statistical analyses were performed with Comprehensive Meta-Analysis software, version 3.0 (Biostat Inc., Englewood, NJ, USA). Pooled estimates and corresponding 95% confidence intervals were calculated for studies reporting on AEs specifically attributable to linezolid. Between-study heterogeneity was evaluated with Cochran’s Q test and the I^2^ statistic, guiding the choice between fixed- and random-effects models. Publication bias was assessed with Begg’s test, with a value of p < 0.05 being considered significant. 

## RESULTS

### Study selection

**Figure 1:**
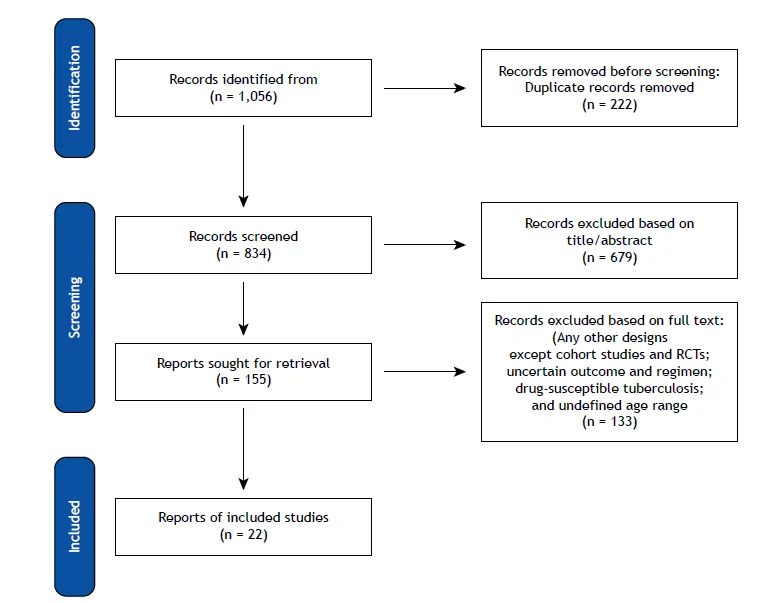
Preferred Reporting Items for Systematic Reviews and Meta-Analyses flow chart of the included studies. RCTs: randomized controlled trials.

As depicted in Figure 1, our comprehensive literature search identified 1,056 records across multiple databases and other sources. After removing 222 duplicates, 834 unique records underwent title and abstract screening, of which 679 were excluded. The remaining 155 full-text articles were assessed for eligibility, resulting in the exclusion of 133 studies. Ultimately, 22 studies reporting on the safety profile of linezolid passed the exclusion criteria and were included in the final analysis.^12^ ^-^ ^33^

### Study characteristics

As summarized in Table 1, a total of 26 study arms published between 2009 and 2025 were included in the final analysis. 

**Table 1:** Characteristics of the included studies.

First Author, year	Study design	Country	No. of patients	No. of patients treated with LZD	Regimens	LZD dose	Duration	LZD duration	Mean age, years	Male	HIV+	BMI	Diabetes status	Smoking	Hepatitis C	DR-TB type
Shukla et al., 2025^12^	RCT	India	25	25	PTO, Z, FQ (MFX/GFX/LFX), PAS, CM/AM, CFZ, CAM, LZD	1,200 mg/day followed by 300-600 mg/day (2 weeks,2 weeks)	NM	4-6 weeks	45	16	0	19.7	5	NM	NM	XDR-TB
Wang et al., 2025^13^	RCT	China	13	13	LZD, BDQ, LFX/MFX, CS, CFZ	1,200 mg/day	2 months	2 months	36.7	8	0	21.2	2	NM	NM	RR/MDR-TB
Jing et al.,2025 (1)*^15^	RCT	China	100	NM	H, E, Z, PTO, LZD, CFZ, MFX	600 mg/day	9-11 months	NM	42.5	70	0	20.2	17	NM	0	MDR-TB
Jing et al., 2025 (2)*^15^	RCT	China	103	NM	BDQ, LZD, CFZ, MFX, CS	600 mg/day	9-11 months	NM	37.5	62	0	20.1	15	NM	0	MDR-TB
Boga et al., 2024^31^	RC	PNG	580	580	LZD-containing regimen	600 mg/day-300 mg/day	562 days	NM	26	303	18	17.5	NM	NM	NM	RR/MDR-TB/Pre-XDR-TB/XDR-TB
Fu et al.,2023 (1)*^18^	PC	China	34	34	BDQ, LZD, MFX, CS, Z	600 mg/day	9-12 months	9-12 months	34.5	25	0	21.3	NM	NM	NM	MDR-TB
Fu et al., 2023 (2)*^18^	PC	China	46	46	LZD, MFX, CS, CFZ, Z	600 mg/day	9-12 months	9-12 months	40	34	0	19.3	NM	NM	NM	MDR-TB
Fu et al., 2023 (3)*^18^	PC	China	24	24	BDQ, LZD, CS, CFZ, Z	600 mg/day	9-12 months	9-12 months	35.5	17	0	20.3	NM	NM	NM	Pre-XDR-TB
Nguyen et al., 2023^28^	PC	Vietnam	106	NM	BDQ, LFX, LZD, CFZ, Z	600 mg/day	9-11 months	NM	41	75	1	NM	NM	NM	NM	RR-TB
Padmapriyadarsini et al., 2022^26^	PC	India	165	NM	BDQ, DLM, LZD, CFZ	600 mg/day-300 mg/day	24-36 weeks	NM	27	92	0	17.3	19	22	0	MDR-TB/Pre-XDR-TB/XDR-TB
Esmail et al., 2022^14^	RCT	South Africa	49	49	BDQ, LZD, LFX, Z, TRD/ETO/high-dose H	600 mg/day	6-9 months	6-9 months	37	34	27	NM	NM	24	NM	MDR/RR-TB
Mishra et al., 2022^30^	RC	India	68	NM	NM	NM	NM	NM	NM	46	NM	NM	NM	NM	NM	MDR-TB
Babu et al., 2021^19^	RC	India	146	NM	H, E, Z, KM, LFX, MFX, ETO, LZD, CS, BDQ, DLM	NM	NM	NM	NM	115	0	NM	NM	NM	NM	NM
Fu et al., 2021^16^	NRCT	China	103	103	LZD, FQ, CS, Z, CFZ, E/PTO, BDQ	NM	9-12 months	9-12 months	38.2	75	NM	20.08	19	39	NM	MDR-TB/RR-TB/Pre-XDR-TB/XDR-TB
McDowell et al., 2021^20^	Descriptive cohort	US	137	137	LZD and tuberculosis treatment	NM	NM	NM	NM	NM	NM	NM	NM	NM	NM	MDR-TB/XDR-TB
Padayatchi et al., 2020^21^	RC	South Africa	151	151	BDQ, LZD, MFX/LFX, PAS, Z, CFZ, CS/TRD, ETO, E, AMX/CLV, CM/KM/AM, H, IPM, CLR	600 mg/day-300 mg/day	24 weeks	24 weeks	33	72	116	NM	NM	NM	NM	Pre-XDR-TB/XDR-TB
Conradie et al., 2020^17^	RCT	South Africa	109	109	BDQ, LZD, PA	1,200 mg/day	26 weeks	26 weeks	35	57	56	19.7	NM	NM	NM	MDR-TB/XDR-TB
Letswee et al., 2019^32^	RC	South Africa	27	27	LZD therapy	600 mg/day	12 months	12 months	36	16	15	NM	NM	NM	NM	MDR-TB/XDR-TB
Jones et al., 2019^22^	RC	South Africa	32	NM	BDQ, CFZ, MFX, LFX, LZD, KM, Z	NM	NM	NM	30	12	16	NM	NM	NM	NM	MDR-TB/XDR-TB
Prajapati et al., 2017^25^	PC	India	112	112	CM, PAS, MFX, high-dose H, CFZ, LZD, AMX/CLV	600 mg/day	27-30 months	27-30	33	83	1	16.79	2	53	NM	XDR-TB
Bolhuis et al., 2015^33^	RC	the Netherlands/Italy	58	58	LZD, tuberculosis treatment regimen	NM	NM	NM	NM	NM	NM	NM	NM	NM	NM	NM
Carrol et al., 2012^23^	PC	South Korea	655	29	H, RFB, RIF, E, Z, KM, STREP, AM, CM, FQs, PAS, CS, PTO, LZD, CLR, AMX/CLV, RXM	NM	NM	NM	NM	551	0	NM	143	NM	NM	NM
Alffenaar et al., 2010^29^	PC	the Netherlands	8	8	LZD, background regimen	600-1,200 mg/day	NM	56 days	28	4	NM	20.8	1	NM	NM	MDR-TB/XDR-TB
Udwadia et al., 2010^27^	PC	India	78	18	NM	1,200 (in 57 patients)-600 mg/day (in 28 patients)	NM	375 days (20.6 months)	29	36	NM	NM	NM	NM	NM	MDR-TB/XDR-TB
Migliori et al., 2009 (1)*^24^	RC	Multicenter	28	28	FQs, SLI, LZD	600 mg/day	7 months	NM	NM	NM	0	NM	NM	NM	NM	MDR-TB/XDR-TB
Migliori et al., 2009 (2)*^24^	RC	Multicenter	57	57	FQs, SLI, LZD	1,200 mg/day	7 months	NM	NM	NM	0	NM	NM	NM	NM	MDR-TB/XDR-TB

(..)*: number of study arms; RC: retrospective cohort study; PC: prospective cohort study; RCT: randomized controlled trial; NRCT: nonrandomized controlled trial; DR-TB: drug-resistant tuberculosis; RR-TB: rifampin-resistant tuberculosis; MDR-TB: multidrug-resistant tuberculosis; XDR-TB: extensively drug-resistant tuberculosis; CAM: complementary and alternative medicine; NM: not mentioned; KM: kanamycin; CS: cycloserine; ETO: ethionamide; BDQ: bedaquiline; LFX: levofloxacin; CFZ: clofazimine; Z: pyrazinamide; E: ethambutol; AM: amikacin; CM: capreomycin; MFX: moxifloxacin; PAS: para-aminosalicylic acid; LZD: linezolid; IPM: imipenem; CLV: clavulanic acid; H: isoniazid; TRD: terizidone; FQs: fluoroquinolones; DLM: delamanid; AMX/CLV: amoxicillin-clavulanic acid; STR: short treatment regimen; RFB: rifabutin; RIF: rifampin; S: streptomycin; CLR: clarithromycin; OBR: optimized background regimen; SLI: second-line injectable drugs; RXM: roxithromycin; PA: pretomanid; PTO: prothionamide; and PNG: Papua New Guinea.

Fifteen studies^12^ ^-^ ^19^ ^,^ ^21^ ^,^ ^22^ ^,^ ^25^ ^-^ ^27^ ^,^ ^30^ ^,^ ^32^ (68.2% of all studies included in the present study) were based in high-burden tuberculosis countries such as China, India, and South Africa, with additional contributions from Vietnam, Papua New Guinea, South Korea, the United States, and several European countries.^20^ ^,^ ^23^ ^,^ ^24^ ^,^ ^28^ ^,^ ^29^ ^,^ ^31^ ^,^ ^33^ Three quarters of the cohort studies included in the final analysis focused on MDR-TB cases, although a substantial proportion also included RR-TB, pre-XDR-TB, and XDR-TB cases. 

Sample sizes varied widely, ranging from 8 to 655 participants per study arm. In most studies, all participants received linezolid as part of their treatment regimen; however, in several studies, only a subset of patients were exposed to linezolid. Background regimens frequently included bedaquiline, clofazimine, and fluoroquinolones, often supplemented with cycloserine and pyrazinamide. Cohorts with older patients and with a more extended resistant profile commonly incorporated injectable agents and second-line drugs such as para-aminosalicylic acid, ethionamide, and terizidone. 

The most frequently administered dose of linezolid was 600 mg daily (N = 15). However, six datasets employed higher doses (1,200 mg/day) or step-down protocols, particularly in response to adverse drug reactions, with dose reductions from 1,200 to 300-600 mg/day. Treatment duration was highly variable, ranging from 4-6 weeks in short-course regimens to 24-26 weeks in standardized bedaquiline, pretomanid, and linezolid (BPaL)/bedaquiline, pretomanid, linezolid, and moxifloxacin (BPaLM) regimens, and up to 9-12 months or longer in earlier XDR-TB cohorts. 

The total number of participants from the included studies was 3,014, of which 2,512 were from 16 cohort studies and 502 from 6 RCTs. On average, study participants were = 34.99 years of age (range, 26-45), and males constituted the majority of the sample (n = 1,803). 

In total, 250 of 3,014 patients were HIV-positive. Coinfection with HIV was reported among all studies that took place in South Africa and providing such data. Outside South Africa, only 20 participants had HIV coinfection, suggesting that detailed reporting of this subgroup may not be necessary. The mean BMI, reported in a subset of studies, was 19.15 kg/m^2^, indicating a high prevalence of underweight status-a potential factor influencing drug pharmacokinetics and tolerability. Data on diabetes mellitus and smoking status were inconsistently reported, with 223 and 138 individuals affected, respectively. 

### Quality assessment

Risk-of-bias appraisal with the Cochrane Collaboration tool indicated overall moderate methodological quality across the six experimental studies included. All trials^12^ ^-^ ^17^ adequately described random sequence generation, which was low risk in five studies and high risk in two. In contrast, allocation concealment and blinding domains consistently demonstrated a high risk of bias. The domains of incomplete outcome data and selective reporting were generally rated low risk, suggesting acceptable data integrity and transparency in outcome reporting (supplementary material, Table S1). 

For the 16 observational cohorts^(18-30,32.33)^ appraised using the Newcastle-Ottawa Scale, methodological quality ranged from moderate to high. Seven studies^18^ ^-^ ^24^ achieved the maximum score of seven. The remaining cohorts^25^ ^-^ ^30^ ^,^ ^32^ ^,^ ^33^ scored 5 points (supplementary material, Table S2). 

### Treatment outcomes of the included studies

**Figure 2:**
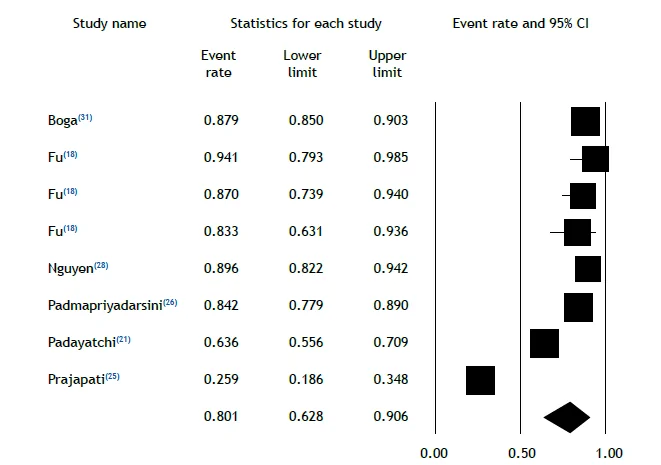
Forest plot summarizing favorable outcomes in cohort studies.

The pooled rate of favorable outcomes (cure or treatment completion) among cohort and clinical trials were 80.1% (95% CI, 62.8-90.6; Figure 2) and 74.6% (95% CI, 54.4-87.9; Figure S1a), respectively. Conversely, the pooled rate of unfavorable outcomes (treatment failure, loss to follow-up, or death) among cohort and clinical trials were 15.4% (95% CI, 6.4-32.7; Figure S1b) and 18.0% (95% CI, 10.4-29.4; Figure S1c), respectively. Detailed results of the pooled analysis are provided in Table S3 and Table 2. Overall, treatment with linezolid-containing regimens demonstrated high efficacy across diverse study settings, although outcome variability likely reflected differences in resistance profiles, background regimens, and patient characteristics. 

**Table 2:** Summary of the different outcomes of the included studies.

Outcomes	Study design	Pooled (95% CI)	Begg	N of patients
Favorable outcomes	Cohort	80.1 (62.8-90.6)	0.71	1,218
	Clinical trails	74.6 (54.4-87.9)	0.45	489
Unfavorable outcomes	Cohort	15.4 (6.4-32.7)	0.71	1,218
	Clinical trails	18.0 (10.4-29.4)	0.80	386
Cured	Cohort	48.0 (22.3-74.8)	0.80	967
	Clinical trails	65.1 (35.5-86.4)	1.00	228
Completed	Cohort	10.3 (1.3-50.4)	1.00	949
	Clinical trails	13.2 (4.5-32.9)	0.29	228
Failure	Cohort	4.8 (2.2-10.1)	0.90	1,071
	Clinical trails	10.7 (5.5-19.6)	1.00	277
LTFU	Cohort	6.8 (3.7-12.2)	0.53	1,071
	Clinical trails	6.9 (2.6-17.1)	0.30	277
Mortality	Cohort	8.7 (3.3-21.1)	0.75	1,250
	Clinical trails	4.0 (1.5-10.2)	0.30	277

LTFU: loss to follow-up.

### AEs

**Figure 3:**
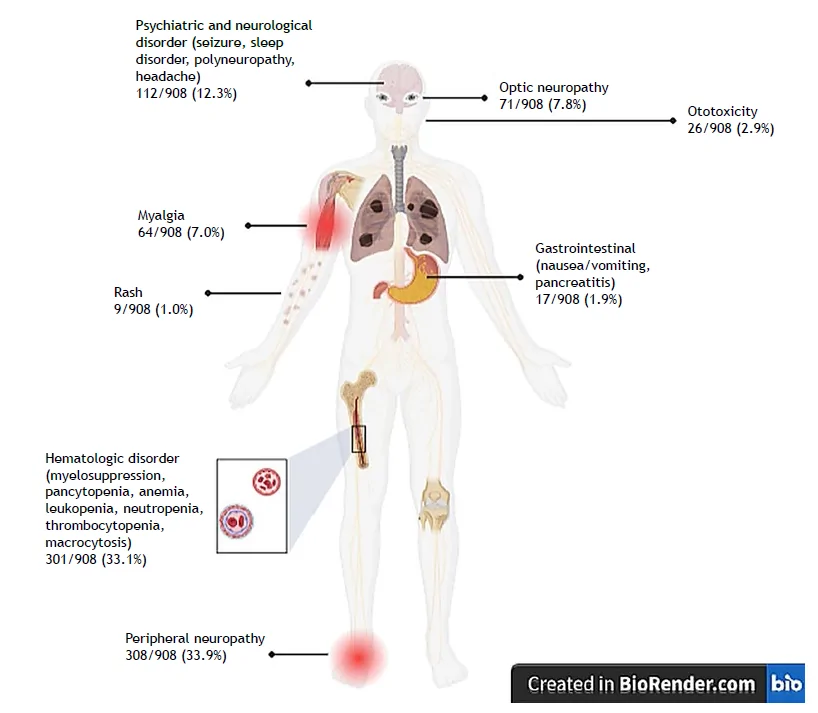
Adverse events associated with linezolid (LZD) in patients with drug-resistant tuberculosis. The figure illustrates the frequency and distribution of major LZD-related adverse effects across different organ systems, including hematologic disorders (33.1%), peripheral neuropathy (33.9%), psychiatric and neurological disorders (12.3%), myalgia (7.0%), optic neuropathy (7.8%), gastrointestinal events (1.9%), ototoxicity (2.9%), and rash (1.0%). Data are expressed as number of events per total of patients (n/N, %). Figure created using BioRender.com

Across the 22 included studies, linezolid exhibited a consistent and characteristic toxicity profile, dominated by hematologic AEs and peripheral neuropathy, followed by gastrointestinal intolerance. Less common but clinically relevant toxicities included optic neuropathy, ototoxicity, and dermatological reactions (Table 3 and Figure 3). 

**Table 3:** Adverse events in the included studies.

First author, year	No. of patients	No. of patients treated with LZD	No. of patients with AEs	No. of serious AEs	Increased QTcF	Hepatic system (%)	Hyperuricemia	Optic neuropathy/ Blurred vision (%)	Ototoxicity/Hearing loss (%)	Hematological system (%)	Gastrointestinal system (%)	Electrolyte disturbance (%)	Psychiatric disorder (%)	skin and mucous membrane (%)	Cardiovascular system (without QTcf) (%)	Peripheral neuropathy	Nervous system (%)	Musculoskeletal disorders (%)
Duration of LNZ treatment < 6 months																		
Shukla et al.^12^	25	25	NM	NM	NM	NM	NM	4	NM	12	11	NM	NM	NM	NM	NM	NM	NM
Wang et al.^13^	13	13	12	NM	NM	NM	NM	NM	NM	4	3	NM	NM	NM	NM	7	NM	NM
Alffenaar et al.^29^	8	8	NM	NM	NM	NM	NM	0	NM	NM	NM	NM	NM	NM	NM	NM	NM	NM
Duration of LNZ treatment = 6-12 months																		
Fu et al., 2023 (1)^18^	34	34	NM	NM	NM	NM	NM	NM	NM	NM	NM	NM	NM	NM	NM	13	24	23
Fu et al., 2023 (2)^18^	46	46	NM	NM	NM	NM	NM	NM	NM	NM	NM	NM	NM	NM	NM	26	39	29
Fu et al., 2023 (3)^18^	24	24	NM	NM	NM	NM	NM	NM	NM	NM	NM	NM	NM	NM	NM	12	15	11
Esmail et al., 2022^14^	49	49	NM	NM	NM	NM	NM	NM	NM	10	NM	NM	NM	NM	NM	1	NM	NM
Fu et al., 2021^16^	103	103	NM	NM	NM	NM	NM	1	NM	1	NM	NM	NM	NM	NM	2	NM	NM
Conradie et al.^17^	109	109	NM	NM	NM	NM	NM	2	NM	NM	NM	NM	NM	NM	NM	NM	NM	NM
Padayatchi et al.^21^	151	151	NM	NM	NM	NM	NM	34	24	70	NM	NM	22	NM	NM	56	8	NM
Letswee et al.^32^	27	27	NM	NM	NM	NM	NM	NM	NM	7	NM	NM	NM	NM	NM	NM	NM	NM
Duration of LNZ treatment > 12 months																		
Prajapati et al.^25^	112	112	NM	NM	NM	NM	NM	NM	NM	3	NM	NM	NM	NM	NM	NM	NM	NM
Udwadia et al.^27^	78	18	NM	NM	NM	NM	NM	1	NM	1	NM	NM	NM	NM	NM	16	NM	NM
Duration unknown																		
Jing et al. (1)^15^	100	NM	NM	NM	NM	NM	NM	NM	NM	13	NM	NM	NM	NM	NM	10	NM	NM
Jing et al. (2)^15^	103	NM	NM	NM	NM	NM	NM	NM	NM	11	NM	NM	NM	NM	NM	26	NM	NM
Boga et al.^31^	580	580	NM	NM	NM	NM	NM	7	NM	NM	NM	NM	NM	NM	NM	NM	NM	NM
Padmapriyadarsini et al.^26^	165	NM	NM	NM	NM	NM	NM	NM	NM	78	NM	NM	NM	NM	NM	69	NM	NM
Nguyen et al.^28^	106	NM	11	7	0	0	NM	1	NM	3	1	0	NM	2	0	3	0	1
Babu et al.^19^	146	NM	NM	NM	NM	NM	NM	NM	NM	1	NM	NM	NM	NM	NM	1	NM	NM
Mishra et al.^30^	68	NM	11	NM	NM	NM	NM	NM	2	NM	NM	NM	NM	NM	NM	3	NM	NM
McDowell et al.^20^	137	137	NM	NM	NM	NM	NM	20	NM	41	2	NM	NM	NM	NM	45	1	NM
Jones et al.^22^	32	NM	NM	NM	NM	NM	NM	NM	NM	1	NM	NM	NM	7	NM	NM	NM	NM
Bolhuis et al.^33^	58	58	NM	NM	NM	NM	NM	1	NM	14	NM	NM	NM	NM	NM	11	NM	NM
Carrol et al.^23^	655	29	8	NM	NM	NM	NM	NM	NM	1	NM	NM	NM	NM	NM	7	NM	NM
Migliori et al. (1)^24^	28	28	NM	NM	NM	NM	NM	NM	NM	3	NM	NM	NM	NM	NM	NM	1	NM
Migliori et al. (2)^24^	57	57	NM	NM	NM	NM	NM	NM	NM	27	NM	NM	NM	NM	NM	NM	2	NM

LZD: linezolid; NM: not mentioned; AEs: adverse events; and QTcF: Fridericia-corrected QT interval.

Hematologic toxicity-primarily anemia, thrombocytopenia, and leukopenia-was the most commonly reported AE. Incidence varied widely across studies, ranging from 1 case^16^ ^,^ ^19^ ^,^ ^22^ ^,^ ^23^ ^,^ ^27^ to 78 cases.^26^. This variability likely reflects differences in treatment duration, linezolid dose, and monitoring practices. 

Peripheral neuropathy was frequently reported, with the number of cases ranging from 1 case^19^ to 16 cases.^27^ The risk of neuropathy was generally higher in cohorts receiving prolonged or high-dose linezolid, highlighting the importance of careful neurological monitoring. 

Gastrointestinal events were common but variable, ranging from 11 cases in Shukla et al.^12^ to 3 cases in Wang et al.,^13^ 2 cases in McDowell et al.,^20^ and 1 case in Nguyen et al*.* ^28^

Optic neuropathy or blurred vision was reported in 34 cases in Padayatchi et al.^21^ and was absent in Alffenaar et al.^29^ Ototoxicity or hearing loss occurred infrequently, with 24 cases in Padayatchi et al.^21^ and 2 cases in Mishra et al.^30^

Electrolyte disturbances were generally absent when monitored. Skin or mucous events were reported sporadically but could be substantial in specific series, with 7 cases in Jones et al.^22^ and 2 cases in Nguyen et al.^28^

Serious AEs were rarely quantified, with 7 cases reported in Nguyen et al.^28^ Fridericia-corrected QT interval prolongation was not observed in any study. 

Of 1,608 patients exposed to linezolid, 230 in 6 study arms received it at the daily dose of 1,200 mg, whereas 1,378 patients in 13 study arms received it at the reduced dose of 600 mg. The AEs reported with the 1,200 mg dose were as follows: seven patients developed optic neuropathy, 44 experienced hematologic toxicity, 14 had gastrointestinal events, 2 had nervous system disorders, and 23 experienced peripheral neuropathy. With the 600 mg dose, the following AEs were reported: 64 had optic neuropathy, 257 had hematologic disorders, 3 had gastrointestinal disorders, 22 had psychiatric disorders, 9 had skin diseases, 285 had peripheral neuropathies, 88 had nervous system disorders, and 64 had musculoskeletal disorders. Because several studies did not report on the administered linezolid dose, precise stratification by dose was not always possible. 

## DISCUSSION

The objective of our systematic review and meta-analysis was to explore the safety and tolerability directly attributable to linezolid in regimens designed to treat RR/MDR-TB in studies published between 2009 and 2025. 

The results of the study clearly show that an important proportion of patients experienced AEs specifically attributed to linezolid at the 600 mg and 1,200 mg doses. 

Hematologic disorders (anemia, thrombocytopenia, and leukopenia, in 33.1%) and peripheral neuropathy (in 33.9%) were the most common AEs, followed by psychiatric and neurological disorders (in 12.3%), myalgia (in 7.0%), optic neuropathy (in 7.8%), gastrointestinal events (in 1.9%), ototoxicity (in 2.9%), and rash (in 1.0%). 

The aforementioned results are consistent with those of previous studies.^2^ ^,^ ^33^ ^,^ ^34^ In fact, AEs from linezolid treatment for tuberculosis are common, occurring in approximately 40% to over 80% of patients, with the highest rates being associated with the 1,200 mg dose and longer treatment duration. Common issues include anemia (in 31-85%), thrombocytopenia (in 6-13.5%), and peripheral neuropathy (in 32-81%). These toxicities can lead to treatment interruptions, dose reductions, or permanent discontinuation.^2^ ^,^ ^34^ ^-^ ^36^ In a retrospective, nonrandomized, unblinded observational study^2^ conducted in four European countries to evaluate the safety and tolerability of linezolid at 600 mg, 41.2% of patients reported major AEs attributed to linezolid (anemia, thrombocytopenia, and/or polyneuropathy), with discontinuation in 77% of cases. In a recent individual patient data meta-analysis,^34^ peripheral neuropathy was one of the most common AEs leading to treatment discontinuation, in 15% of patients. Of note, serious AEs were rarely reported. 

One study^2^ demonstrated a high frequency (40.4%) of major AEs associated with linezolid, especially at 1,200 mg. The prevalence of serious AEs in the present study was likely lower than that reported when evaluating the whole regimen because it is difficult to attribute the cause of the AEs to linezolid, given that they may be due to other drugs. In a recent meta-analysis,^34^ it was shown that serious AEs were relatively uncommon among participants using BPaL regimens. 

We observed a higher proportion of AEs with the 600 mg dose. This is likely due to a higher number of studies prescribing 600 mg. Moreover, since AEs were not categorized by severity, similar events of differing intensity may have occurred across the two dose groups. The clinical approach reported by the authors when minor AEs occurred at the higher doses of linezolid was a dose reduction, whereas, in cases of major AEs, treatment with linezolid was usually discontinued. 

There is growing literature that questions the “simple” direct correlation between AEs and linezolid dose; it is suggested that the correlation is rather between AEs and linezolid exposure.^37^ The efficacy and toxicity of linezolid are determined by the exposure achieved in the patient; numerous clinical and population pharmacokinetics studies have identified threshold measurements for both parameters.^38^ On the basis of the findings of reviews, it may be beneficial for future studies to consider incorporating therapeutic drug monitoring into treatment protocols for linezolid. Therapeutic drug monitoring of linezolid is feasible, and models have been developed.^39^ ^,^ ^40^ Therapeutic drug monitoring can help modify the dosing of linezolid to individual patients, particularly those at a higher risk of AEs, thereby enhancing safety and efficacy. In settings where technological and/or economic resources are available, therapeutic drug monitoring can be a tool to personalize the linezolid dose when AEs occur.^41^ Monitoring drug levels could be particularly valuable in minimizing dose-dependent toxicities. Future research could also explore the development of new drug formulations with more favorable safety profiles, in conjunction with therapeutic drug monitoring, to further improve patient outcomes. 

In a meta-analysis including three clinical trials with BPaL-containing regimens,^34^ AEs were shown to be more common in groups randomized to higher doses of linezolid; 35% of patients randomized to a daily dose of 1,200 mg and 22% of those randomized to 600 mg of linezolid experienced a grade 3-4 AE. In another study,^2^ the frequency of serious AEs was found to be higher with a dose of 1,200 mg of linezolid in treatments longer than 8 weeks, with 31.8% requiring discontinuation of the drug. Despite these differences in AEs with the different doses, studies have shown no differences in outcomes. In the present meta-analysis, efficacy was high across diverse study settings, with a pooled rate of favorable outcomes (cure or treatment completion) of 77.8%. In another study,^2^ the efficacy endpoints (sputum and culture conversion, or positive treatment outcome) were similar with 600 mg and 1,200 mg of linezolid. 

The limitation of our systematic review and meta-analysis is related to the limitations of the included studies, especially their heterogeneity. Despite this limitation, the present study assessed the safety and tolerability directly attributed to linezolid rather than the regimen as a whole, as was done in most of the previous studies. Future studies on drugs need to include a clear description of safety and tolerability, specifying regimen, dose, and timing of administration for each drug, as well as regimen duration, together with a complete description of AEs and serious AEs as per the WHO Active TB drug-safety monitoring and management recommendations, in order to allow comparative analysis and empower meta-analyses.^2^ ^,^ ^42^

In conclusion, the results of our study clearly show that linezolid causes frequent and severe AEs, with over one third of patients presenting with documented hematologic disorders (including anemia, thrombocytopenia, and leukopenia) and peripheral neuropathy, and more than 12% presenting with documented psychiatric and neurological disorders. 

Although the recommended linezolid-containing regimens are considered safe, linezolid still causes frequent and severe AEs. New drugs with similar characteristics but lower toxicity will significantly improve the safety of antituberculosis regimens, with improved adherence and treatment outcomes. 
